# Human brown adipose tissue [^15^O]O_2_ PET imaging in the presence and absence of cold stimulus

**DOI:** 10.1007/s00259-016-3364-y

**Published:** 2016-03-19

**Authors:** Mueez u Din, Juho Raiko, Teemu Saari, Nobu Kudomi, Tuula Tolvanen, Vesa Oikonen, Jarmo Teuho, Hannu T Sipilä, Nina Savisto, Riitta Parkkola, Pirjo Nuutila, Kirsi A. Virtanen

**Affiliations:** 1 Turku PET Centre, Turku University Hospital, Kiinamyllynkatu 4-8, 20520 Turku, Finland; 2 Turku PET Centre, University of Turku, Kiinamyllynkatu 4-8, 20520 Turku, Finland; 3 Department of Medical Physics, Faculty of Medicine, Kagawa University, Kagawa, Japan; 4 Department of Radiology, Turku University Hospital and University of Turku, Turku, Finland

**Keywords:** Brown adipose tissue, cold-induced thermogenesis, oxygen consumption, [^15^O]O_2_ PET imaging

## Abstract

**Purpose:**

Brown adipose tissue (BAT) is considered a potential target for combatting obesity, as it produces heat instead of ATP in cellular respiration due to uncoupling protein-1 (UCP-1) in mitochondria. However, BAT-specific thermogenic capacity, in comparison to whole-body thermogenesis during cold stimulus, is still controversial. In our present study, we aimed to determine human BAT oxygen consumption with [^15^O]O_2_ positron emission tomography (PET) imaging. Further, we explored whether BAT-specific energy expenditure (EE) is associated with BAT blood flow, non-esterified fatty acid (NEFA) uptake, and whole-body EE.

**Methods:**

Seven healthy study subjects were studied at two different scanning sessions, 1) at room temperature (RT) and 2) with acute cold exposure. Radiotracers [^15^O]O_2_, [^15^O]H_2_O, and [^18^F]FTHA were given for the measurements of BAT oxygen consumption, blood flow, and NEFA uptake, respectively, with PET-CT. Indirect calorimetry was performed to assess differences in whole-body EE between RT and cold.

**Results:**

BAT-specific EE and oxygen consumption was higher during cold stimulus (approx. 50 %); similarly, whole-body EE was higher during cold stimulus (range 2–47 %). However, there was no association in BAT-specific EE and whole-body EE. BAT-specific EE was found to be a minor contributor in cold induced whole-body thermogenesis (almost 1 % of total whole-body elevation in EE). Certain deep muscles in the cervico-thoracic region made a major contribution to this cold-induced thermogenesis (CIT) without any visual signs or individual perception of shivering. Moreover, BAT-specific EE associated with BAT blood flow and NEFA uptake both at RT and during cold stimulus.

**Conclusion:**

Our study suggests that BAT is a minor and deep muscles are a major contributor to CIT. In BAT, both in RT and during cold, cellular respiration is linked with circulatory NEFA uptake.

**Electronic supplementary material:**

The online version of this article (doi:10.1007/s00259-016-3364-y) contains supplementary material, which is available to authorized users.

## Introduction

Brown adipose tissue (BAT) provides non-shivering thermogenesis (NST) because of the presence of the mitochondrial uncoupling protein-1(UCP-1) [1] and BAT activity remains present in humans in adulthood [2–4]. Cold exposure stimulates the sympathetic nervous system (SNS), which triggers BAT into heat production [5].

Previous studies showing metabolically active BAT under cold stress are based on high symmetrical bilateral [^18^F]FDG uptake in supraclavicular adipose tissue regions [6–9], while active BAT is considered to rely primarily on fatty acids as the major fuel for thermogenesis [10]. The study by Muzik et al. [11] has shown that during cold stress higher [^18^F]FDG standardised uptake values (SUVs) correlate with higher oxygen consumption in BAT. However, a proportional glucose contribution as a BAT substrate could not be suggested in this study due to the static [^18^F]FDG PET measurements. Moreover, BAT-specific energy expenditure (EE) during cold is unclear [12], while the study by Muzik et al. [11] suggests BAT to be a minor contributor to cold induced thermogenesis. Therefore, it was critical to confirm BAT-specific EE using direct oxygen consumption measurements by using [^15^O]O_2_ positron-emission tomography (PET) imaging. Moreover, it has also not yet been established how much of the BAT circulatory NEFA uptake is associated with BAT-specific thermogenesis.

In the present study, we hypothesized that increased oxygen consumption by brown adipocytes under cold stimulation can be identified using in vivo [^15^O]O_2_ PET imaging in human supraclavicular BAT depots, and we further aimed to determine whether BAT-associated EE correlates with whole-body EE and additionally with BAT blood flow and NEFA uptake in room temperature (RT) conditions and during cold stimulus.

## Material and methods

### Study subjects

Healthy study subjects (n = 7) of both genders with normal oral glucose tolerance test and cardiovascular status were included after written informed consent and screening. Subjects were screened for hypertension, diabetes, and elevated hepatic enzymes (ALAT, AFOS, GT). Whole-body insulin sensitivity (M-value) was also measured using a hyperinsulinemic euglycemic clamp technique [13]. The basic characteristics are given in Table 1. The local ethical review committee of the Southwestern Finland Hospital District reviewed and approved the study.

**Table 1:** Characteristics of study subjects

Age	years	36 ± 11
Gender	M/F	5 / 2
BMI	kg/m^2^	25.5 ± 3.3
Waist	cm	90.4 ± 10.4
M-value*	mg/kg/min	6.9 ± 3.7
Percentage fat	%	24.8 ± 9.1
Body surface area	m^2^	2.0 ± 0.2

*M-value describes whole body insulin sensitivity

Abbreviations: BMI: Body mass index

Data is shown as mean ± SD

### Study design

**Fig. 1:**
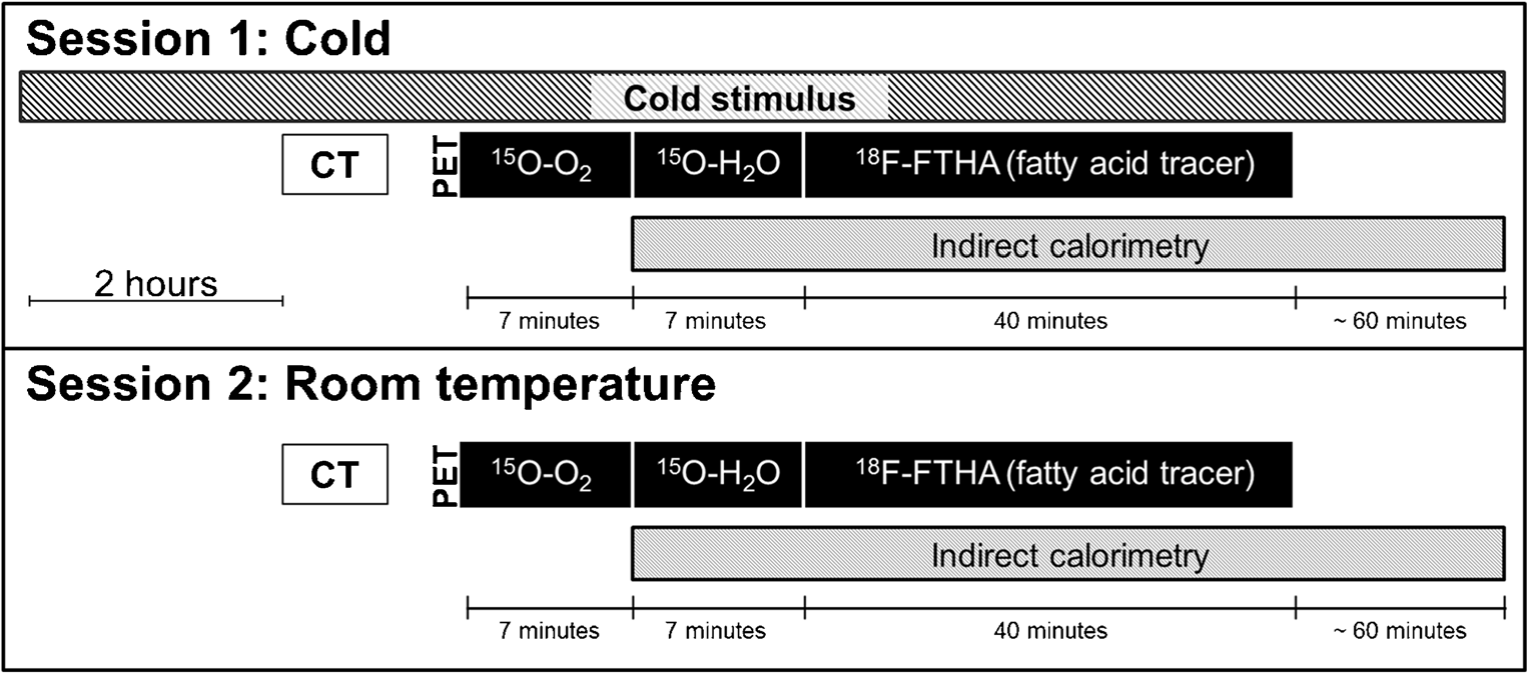
Diagrammatic representation of the study design and scanning protocol utilised to measure whole-body energy expenditure, oxidative metabolism, blood flow, and NEFA uptake at room temperature and during cold stimulus. Abbreviations: CT, X-ray computed tomography; [^15^O]O_2_, Radioactive O^15^ labelled oxygen gas; [^15^O]H_2_O, Radioactive O^15^ labelled water

Subjects underwent two PET scanning sessions; one of the scanning sessions was performed at room temperature and the other during acute cold exposure (Fig. 1). Scanning sessions were organised on separate days in a random order with the minimum interval between the sessions being one week. Studies were performed after overnight fasting. All scans were performed at the same time of day in order to minimise any possible effect caused by individual circadian rhythm. Cold exposure was started 2 h prior to the scan using cooling blankets (Blanketrol III, Cincinnati Sub-Zero, Cincinnati, OH, USA), and cooling was continued during the PET scanning. Cooling was initiated with the temperature of the water circulating in the cooling blanket set to 6 °C; this temperature was gradually raised once the subjects were visually observed to be shivering or reported shivering themselves. The skin temperature of the subjects was also monitored during scanning using a digital thermometer (Art.183, Termometerfabriken Viking AB, Eskilstuna, Sweden) while the temperature sensing probe attached to the lateral abdominal skin surface. RT was maintained at approximately 22 °C.

### Scanning protocol

Subjects were placed supine in a head first position inside the PET-CT scanner (Discovery 690 PET-CT scanner; General Electric Medical Systems, Milwaukee, WI, USA; PET voxel size = 3.64 x 3.64 x 3.27 mm), while the level of the clavicles was set to be the centre of the axial field of view (AFOV). Comfortable, relaxed position of the subjects was ensured in order to avoid any tension in the neck muscles, and the arms were placed next to the body. The positioning of the subjects within the scanner was kept identical irrespective the cooling protocol utilised. Scanning started with an attenuation correction transmission CT scan followed by three separate dynamic emission PET scans using three different radiotracers, i.e. [^15^O]O_2_, [^15^O]H_2_O, and [^18^F]FTHA. In the [^15^O]O_2_ scans, the subjects were given radioactive oxygen gas (509 ± 37 MBq) using a plastic mask with a single deep inhalation and scanning was started simultaneously; 20 frames of variable lengths were acquired over a period of 7 min (6 × 5 s, 6 × 15 s, 6 × 30 s, 2 × 60 s). After sufficient radioactive decay of [^15^O]O_2_ (approx. 10 min.), radiowater [^15^O]H_2_O (493 ± 35 Mbq) was intravenously injected into the left antecubital vein and scanning started immediately using 20 frames with a dynamic acquisition protocol (6 × 5 s, 6 × 15 s, 6 × 30 s, 2 × 60 s). [^18^F]FTHA scans were performed and quantified as described by Saari et al. [14]. Each subject received an estimated radiation dose of 8.8 mSv from the PET-CT scans during our study. The details of the production of tracers and PET image reconstruction can be found in the supplementary data.

### PET image analysis

**Fig. 2:**
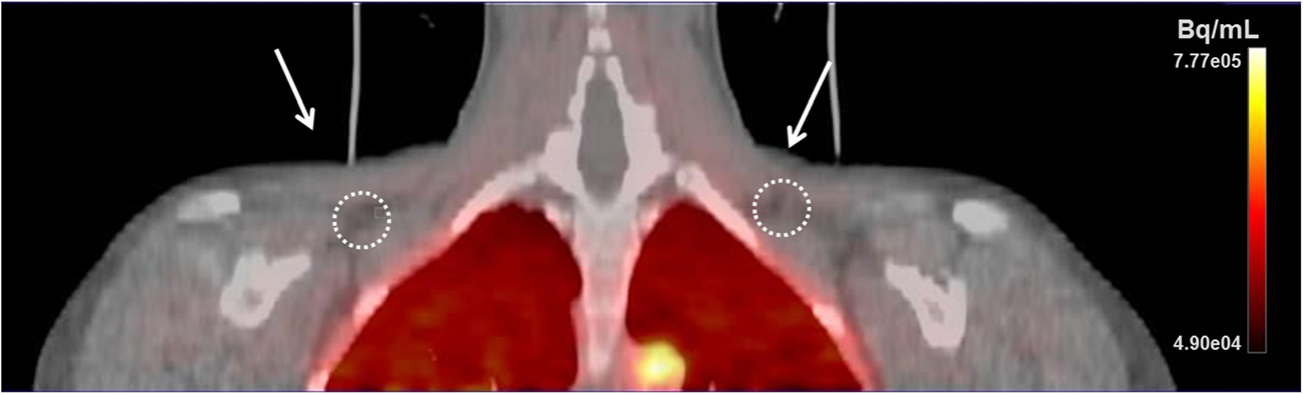
Fused [^15^O]O_2_ PET-CT scan showing the location of three-dimensional VOIs for BAT in supraclavicular fat depots (white circles). Subjects were asked to inhale [^15^O] labelled O_2_; therefore, high radioactive concentration (red colour) can be seen in the lungs region. Arterial input functions were calculated by drawing a VOI on the arch of the aorta (high radioactive concentration can be seen as a yellow bright spot)

Carimas 2.8 software (Turku PET Centre, Turku, Finland) was used to analyse all acquired PET-CT images. The volume of interests (VOIs) in BAT were drawn manually on the supra-clavicular fat depots on the fused PET-CT images by taking into account the CT Hounsfield unit (HU) value of the voxels within −50 to −250 HU range (Fig. 2). White adipose tissue VOIs were drawn on the posterior subcutaneous neck area. For skeletal muscle, VOIs were drawn on deltoid, trapezius, levator scapulae, splenius cervicis, and pectoralis major muscles. For all the radiotracers used, arterial input function was determined by drawing a VOI comprising of 70–100 voxels on the arch of the aorta on the fused PET-CT images.

### Measurement of blood flow (BF) and metabolic rate of oxygen (MRO_2_)

Blood flow was calculated from [^15^O]H_2_O PET scans by assuming a one-tissue compartmental model as follows,

$$ {C}_T(t)={K_1}^W\cdot {C}_A(t)\otimes {e}^{-K2\cdot t}+{V}_A\cdot {C}_A(t) $$

where *C*_T_ is the tissue time activity curve, *C*_A_ is the input function and *V*_A_ is the arterial blood volume. The *K*_1_^w^, *k*_2_, and *V*_A_ values were estimated by an optimization procedure (Gauss-Newton method).

The metabolic rate of oxygen was calculated by incorporating the TACs of [^15^O]O_2_ PET scans by assuming a one-tissue compartmental model as follows,

$$ {C}_T(t)={K_1}^O\cdot {C_A}^O(t)\otimes {e}^{-K2\cdot t}+{K_1}^W\cdot {C_A}^W(t)\otimes {e}^{-K2\cdot t}+{V}_0\cdot {C_A}^0(t) $$

where *C*_*T*_ is the tissue TAC, *C*_*A*_^*O*^ and *C*_*A*_^*w*^ are the input functions for oxygen and water, respectively. For the estimation of oxygen and water content, the aorta TAC was separated for each component according to the mathematical model described by Iida et al. [15]. *V*_0_ is the arterial blood volume. The first term in the equation expresses the kinetics of oxygen and the second that of water, namely recirculation water. The *K*_1_^o^, and *V*_0_ values were estimated by an optimization procedure (Gauss-Newton method), by inputting the obtained *K*_1_^w^ and *k*_2_ from the water data [16]. Subsequently, the MRO_2_ in the specific tissue was calculated as a product of *K*_1_^o^ and the arterial oxygen concentration ^a^O_2_ (mLO_2_/100 mL)_._ The arterial concentration of oxygen was considered to be 19.8 mL per 100 mL of blood volume. MRO_2_ for muscles was calculated by taking into account the oxygen binding in myoglobin [17, 18].

### Indirect calorimetry

In order to measure whole-body EE and substrate utilisation rates, indirect calorimetry (using Deltatrac II, Datex-Ohmeda) was performed simultaneously with PET scans (100–120 min, Fig. 1). From the data set, measurements were excluded from analyses if they deviated more than 1.5 SD from the mean vO_2_, vCO_2_, EE or respiratory quotient values, caused by irregular breathing. The first 30 min of the calorimetry data was also excluded. Whole-body EE, substrate utilisation rates, and respiratory quotient were calculated according to Weir equation [19] and the manufacturer’s equations [20] using Matlab (Version: R2011a). Protein oxidation was accounted for in the equations by considering urinary nitrogen to be 13 g/24 h.

### Tissues mass calculation

BAT mass in the cervico-upper thoracic region was estimated on fused PET-CT images by first thresholding all CT voxels between a range of −50 to −250 HU at all potential cervico-upper thoracic BAT sites (cervical, supraclavicular, and axillary adipose depots). The acquired voxels underwent further thresholding and all voxels with less than 0.7 μmol/100 g/min NEFA uptake on parametric cold-exposure [^18^F]FTHA PET images were excluded. Finally, the volume of all these voxels (cm^3^) was converted into mass by assuming the density of BAT to be 0.92 g/cm^3^. Muscle mass in cervico-upper thoracic region was calculated from the CT images by thresholding all CT voxels at all muscle sites between 0 to +250 HU. Afterwards, the volume of all these voxels (cm^3^) was converted into mass by assuming the density of muscle to be 1.06 g/cm^3^.

### Tissue specific DEE calculation

Finally, tissue specific daily energy expenditure (DEE) was calculated from MRO_2_ according to the formula below,

$$ DEE\; tissue\left( kcal/ day\right)=MR{O}_2\left( mL/100g/ \min \right)\times tissue\; mass(100g)\times 0.0048\left( kcal/ mL\right)\times 1440\left( min/ day\right) $$

The energy *(kcal)* produced per millilitre of oxygen consumption was assumed to be for respiratory quotient (RQ) of 0.80 (4.801 kcal/ litre O_2_ consumed) [21].

### Statistical analyses

Statistical analyses were performed using IBM SPSS Statistics (version 22). To test for differences in mean values, a two-tailed paired Student t-test and a Wilcoxon rank-sum test were used. Pearson and Spearman’s correlation tests were used to analyse correlations. p-value of ≤ 0.05 was considered to be significant.

## Results

### Whole-body energy expenditure

**Fig. 3:**
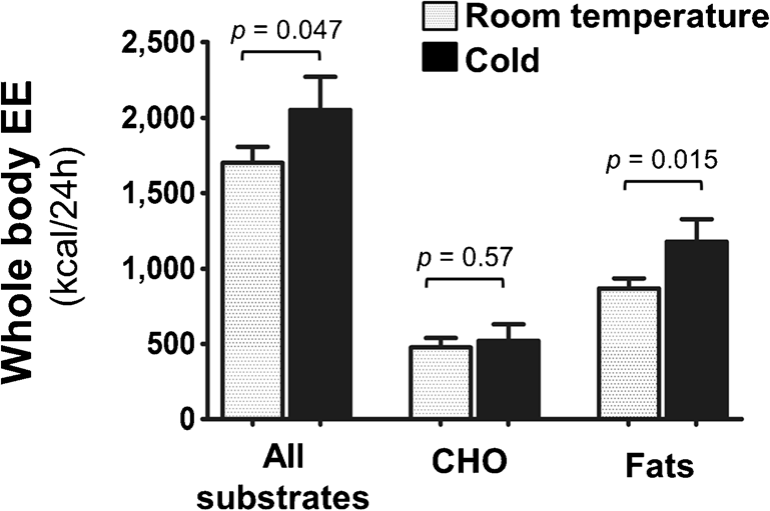
Whole-body EE (kcal/24 h) measured with indirect respiratory calorimetry was significantly higher during cold stress. Whole-body carbohydrate oxidation was unchanged as a result of cold stress; rather, whole-body fat oxidation was significantly elevated. The result has been shown as mean ± SEM. Abbreviation: CHO, carbohydrates; EE, energy expenditure

Whole-body EE was significantly higher (range 2–47 %) after cold stress as compared with RT (1701 ± 282 versus 2052 ± 574 kcal/24 h, *p* = 0.046, Fig. 3); likewise there was also significant increase in whole-body oxygen consumption (vO_2_) during cold stimulus (RT: 248.3 ± 40.2 versus cold: 299.6 ± 82.3 mL/min, *p* = 0.04 – Table 2). The respiratory quotient (RQ) was not significantly influenced by cold stress (Table 2); however, when whole-body EE was divided into subcomponents, we observed that the whole-body fat oxidation was significantly higher during cold stress, while no difference was seen in whole-body carbohydrate oxidation (Fig. 3).

**Table 2:** Differences in measured values in room temperature and cold

Parameter		Room Temp.	Cold	p-value
Whole-Body EE	kcal/24 h	1701 ± 282	2052 ± 574	0.047
RQ		0.81 ± 0.02	0.79 ± 0.03	0.06
vO_2_	mL/min	248.3 ± 40.2	299.6 ± 82.3	0.04
vCO_2_	mL/min	200.2 ± 34.0	238.2 ± 67.4	0.06
Average skin temperature	°C	35.3 ± 0.8	32.4 ± 0.8	<0.0001
BAT NEFA uptake rate	(μmol/100 g/min)	0.8 ± 0.8	1.3 ± 1.3	0.09*
WAT NEFA uptake rate	(μmol/100 g/min)	0.3 ± 0.1	0.3 ± 0.1	0.60
Deltoid muscle NEFA uptake rate	(μmol/100 g/min)	0.5 ± 0.3	0.5 ± 0.3	0.95
Trapezius muscle NEFA uptake rate	(μmol/100 g/min)	0.4 ± 0.2	0.6 ± 0.3	0.13*
Levator scapulae muscle NEFA uptake rate	(μmol/100 g/min)	0.5 ± 0.2	0.7 ± 0.3	0.04*
Splenius cervicis muscle NEFA uptake rate	(μmol/100 g/min)	0.6 ± 0.3	0.7 ± 0.3	0.29
Pectoralis major muscle NEFA uptake rate	(μmol/100 g/min)	0.4 ± 0.2	0.8 ± 0.3	0.06
Plasma Insulin	mU/L	7.1 ± 8.1	4.8 ± 2.8	0.46
Free plasma T3	pmol/L	4.5 ± 0.4	4.7 ± 0.3	0.19
Free plasma T4	pmol/L	14.0 ± 2.6	14.0 ± 2.1	1.0
Plasma TSH	mU/L	1.6 ± 0.6	1.7 ± 0.6	0.21
Plasma Triglyceride	mmol/L	0.8 ± 0.3	1.1 ± 0.4	0.03
Plasma glucose	mmol/L	5.2 ± 0.3	5.2 ± 0.2	0.84

Abbreviations: EE, Energy expenditure; RQ, Respiratory Quotient;

T3, Triiodothyronine; T4, Thyroxine; TSH, Thyroid-stimulating hormone

vO _2_, volume of whole body oxygen consumption; vCO _2_, volume of whole body carbon dioxide production

Data are shown as mean ± SD *Wilcoxon rank-sum test

### Tissue specific metabolic rate of oxygen (MRO_2_) and blood flow (BF)

**Fig. 4:**
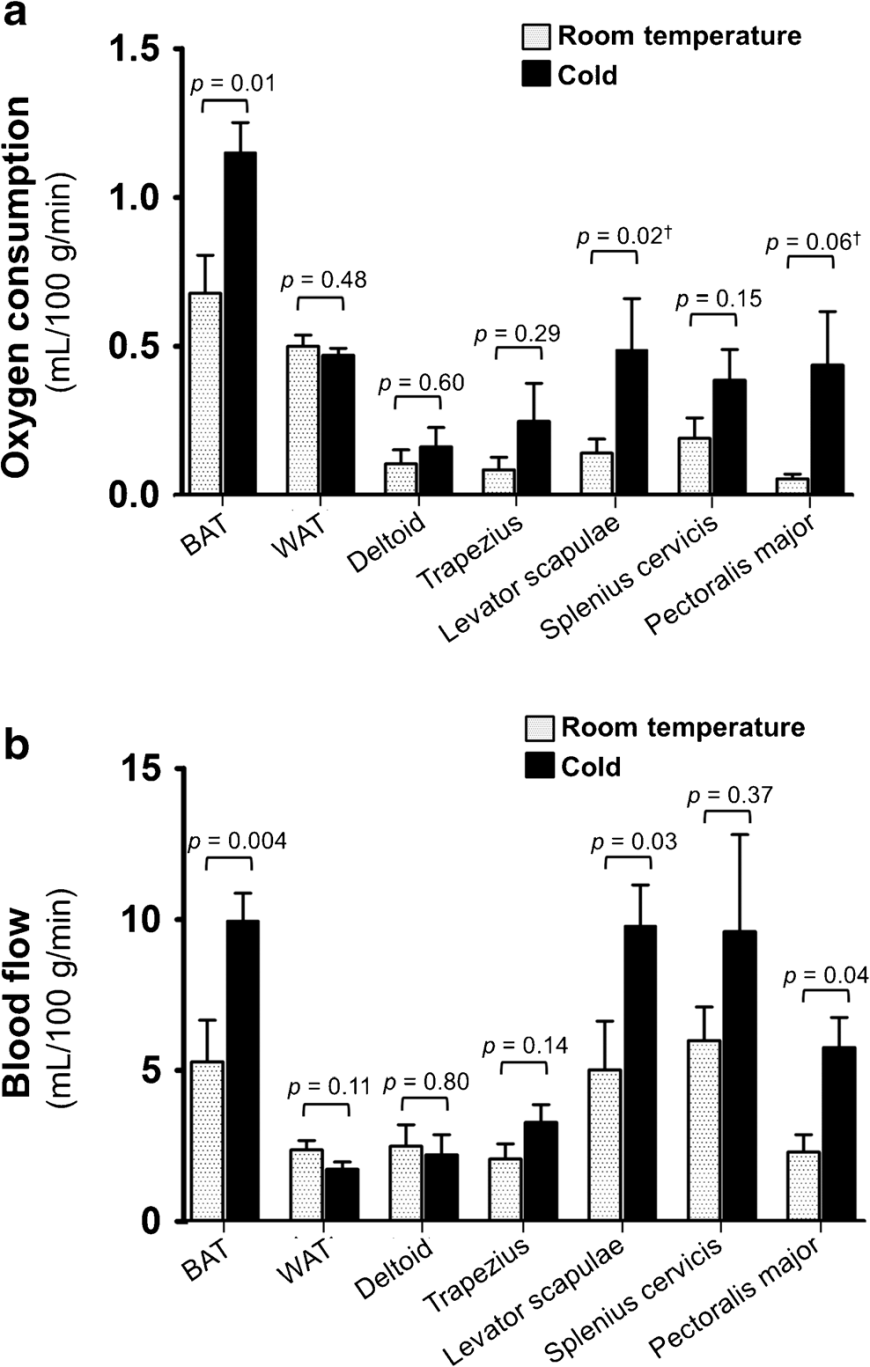
**a**: Metabolic rate of oxygen consumption (MRO_2_) of BAT, WAT and different cervico-thoracic muscles during room temperature and cold, calculated from [^15^O]O_2_ PET scans; results are shown as means ± SEM. **b**: Blood flow in BAT, WAT, and additional muscles during room temperature and cold calculated using [^15^O]H_2_O PET scans; results are shown as means ± SEM. ^Ϯ^ Wilcoxon rank-sum test

Our PET imaging results revealed that the metabolic rate of oxygen (MRO_2_) in BAT during cold stimulus was significantly higher when compared to RT (0.7 ± 0.3 versus 1.2 ± 0.3 mL/100 g/min, *p* = 0.01, RT vs. cold, respectively). The MRO_2_ in the levator scapulae muscle was also higher during cold stress (0.1 ± 0.1 compared to 0.5 ± 0.5 mL/100 g/min, *p* = 0.02, RT vs. cold, respectively). There was a positive trend of increase in MRO_2_ in the pectoralis major (RT: 0.05 ± 0.04 versus cold: 0.4 ± 0.5 mL/100 g/min, *p* = 0.06) and splenius cervicis muscles (RT: 0.2 ± 0.2, cold: 0.4 ± 0.3 mL/100 g/min, *p* = 0.15) while there was no difference in MRO_2_ in the deltoid muscle (0.1 ± 0.1 versus 0.2 ± 0.2 mL/100 g/min, *p* = 0.60, RT vs. cold, respectively), trapezius muscle (0.1 ± 0.1 versus 0.2 ± 0.3 mL/100 g/min, *p* = 0.29) or neck subcutaneous white adipose tissue (0.5 ± 0.1 versus 0.5 ± 0.1 mL/100 g/min, *p* = 0.48, RT vs. cold, respectively, Fig. 4a).

Similarly, blood flow was significantly higher (almost 2-fold) in response to cold in BAT (RT: 5.3 ± 3.7 versus cold: 10.0 ± 2.4 mL/100 g/min, *p* = 0.004), levator scapulae muscle (RT: 5.0 ± 4.2 compared to cold: 9.7 ± 3.5 mL/100 g/min, *p* = 0.03), and pectoralis major muscle (RT: 2.3 ± 1.5 compared to cold: 5.8 ± 2.6 mL/100 g/min, *p* = 0.04). There was a positive trend of high blood flow in the trapezius muscle (RT: 2.0 ± 1.3 compared to cold: 3.2 ± 1.6 mL/100 g/min, *p* = 0.14), while blood flow to the deltoid muscle (RT: 2.5 ± 1.9 versus cold: 2.2 ± 1.8 mL/100 g/min, *p* = 0.80), and subcutaneous neck white adipose tissue (RT: 2.4 ± 0.8 versus cold: 1.7 ± 0.6 mL/100 g/min, *p* = 0.11, Fig. 4b) was not significantly different during cold stress.

We found an inverse relation between BMI and BAT MRO_2_ during cold stress (*rho* = −0.78; *p* = 0.04); moreover, there was also a trend for high radiodense BAT depots (CT radiodensity measured in Hounsfield units) to have higher MRO_2_ during cold stress (*r* = 0.68, *p* = 0.08).

### BAT mass and tissue-associated daily energy expenditure

**Fig. 5:**
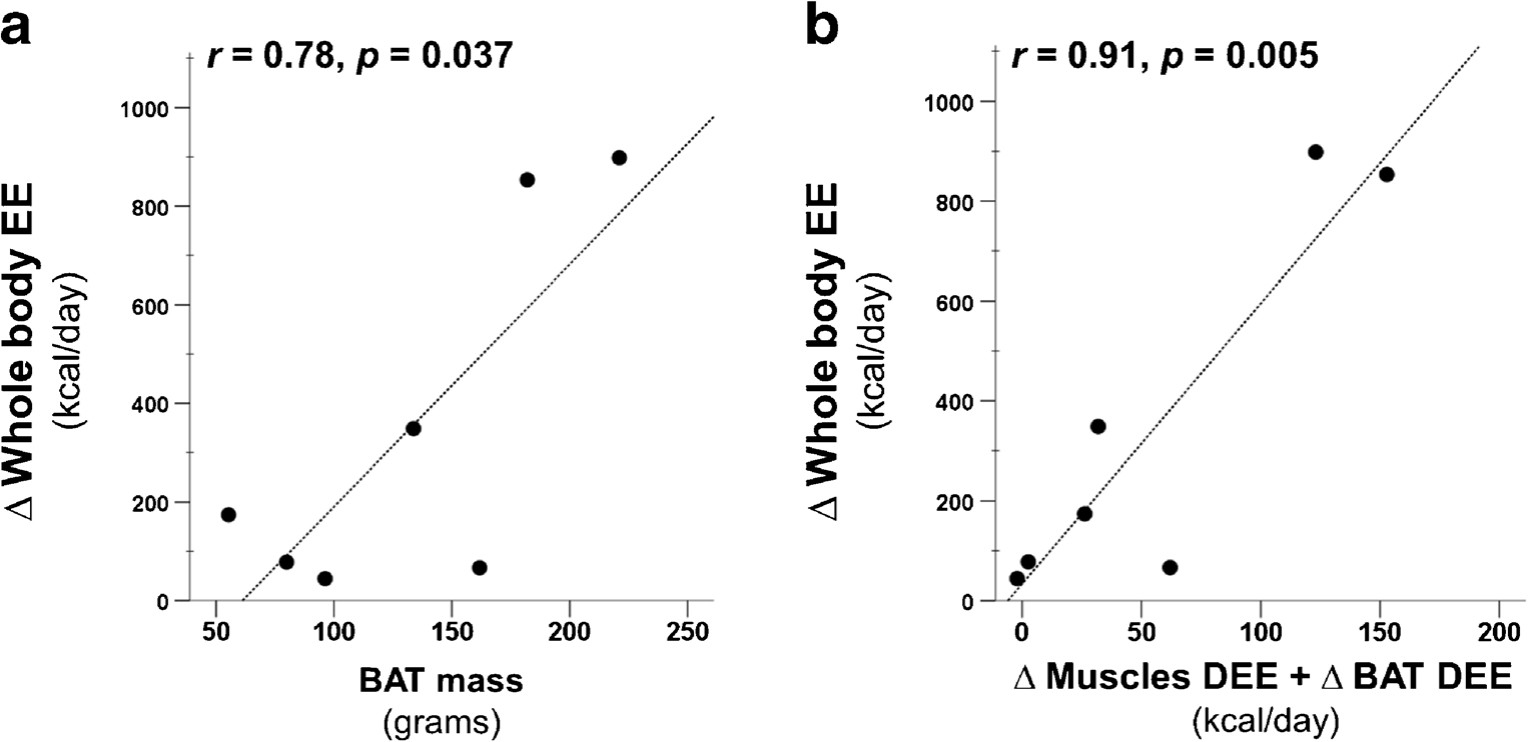
The change (∆) in whole body energy expenditure (EE) during RT and cold stress, measured with indirect calorimetry, was found to be directly correlating with BAT mass (**a**) and also with the sum of the change (∆) in the cervico-upper thoracic muscle-associated daily energy expenditure (DEE) and ∆ BAT-associated DEE, measured with [^15^O]O_2_ PET imaging (**b**)

BAT masses in the cervico-upper thoracic region were highly varied in our study population with a mean of 133 ± 59 grams (range: 55–221 grams). BAT-associated daily energy expenditure (DEE, from MRO_2_ and BAT mass data), was estimated to be 7 ± 5 kcal/day at RT compared to 10 ± 5 kcal/day in cold stress (*p* = 0.02). Change (∆) in BAT DEE as a result of cold stress was estimated to be 4 ± 3 kcal/day; this accounted for merely 1 % of the total change (∆) in whole-body EE (352 ± 372 kcal/day, a range of 45–898 kcal/day). Moreover, we did not find any obvious direct association between whole-body EE and BAT-associated DEE both at RT (*rho* = 0.10, *p* = 0.81) and during cold stress (*rho* = 0.21, *p* = 0.64). Paradoxical to earlier findings, we observed significant association between the change (∆) in whole-body EE (cold induced thermogenesis) and the BAT mass (*r* = 0.78; *p* = 0.037, Fig. 5a). We further looked DEE contribution of the cervico-upper thoracic muscles as a means of explaining the high change (∆) in whole-body EE, by taking into account muscles MRO_2_ (average of all analysed muscles, Fig. 4a) and muscle mass. We estimated that the muscles in the cervico-thoracic region (in our FOV, 15 cm) contributed 33 ± 28 kcal/day (range 11–82 kcal/day) during RT and 86 ± 68 kcal/day (range 11–205 kcal/day, *p* = 0.058) during cold stress. The change (∆) in the DEE of the cervico-thoracic muscles also correlated with the change in whole-body EE (*r* = 0.89, *p* = 0.007). When the change in the BAT DEE was also taken into account along with the change in muscles DEE, the correlation improved (*r* = 0.91, *p* = 0.005, Fig. 5b).

**Fig. 6:**
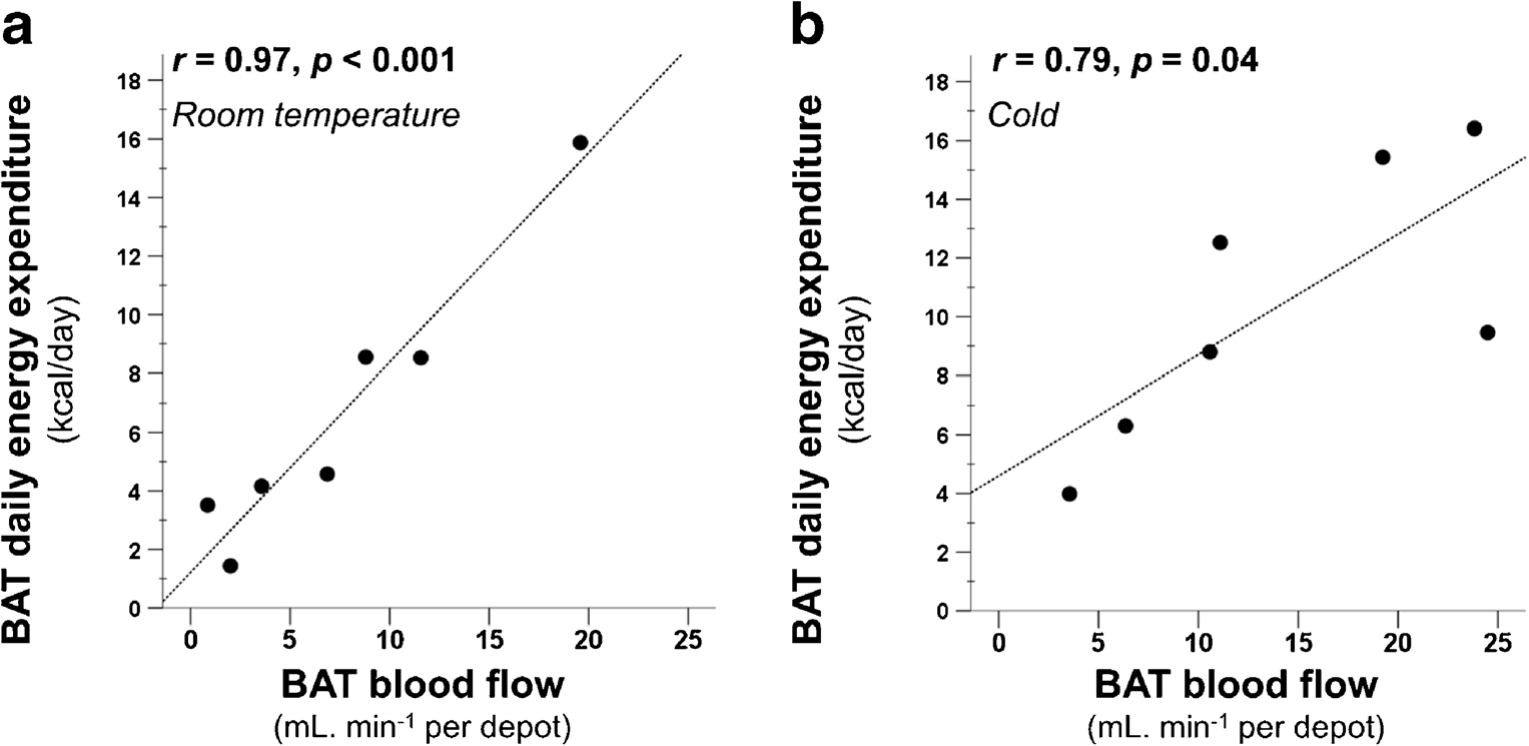
BAT-associated DEE correlated with BAT blood flow both at room temperature (**a**) and also during cold stimulus (**b**)

**Fig. 7:**
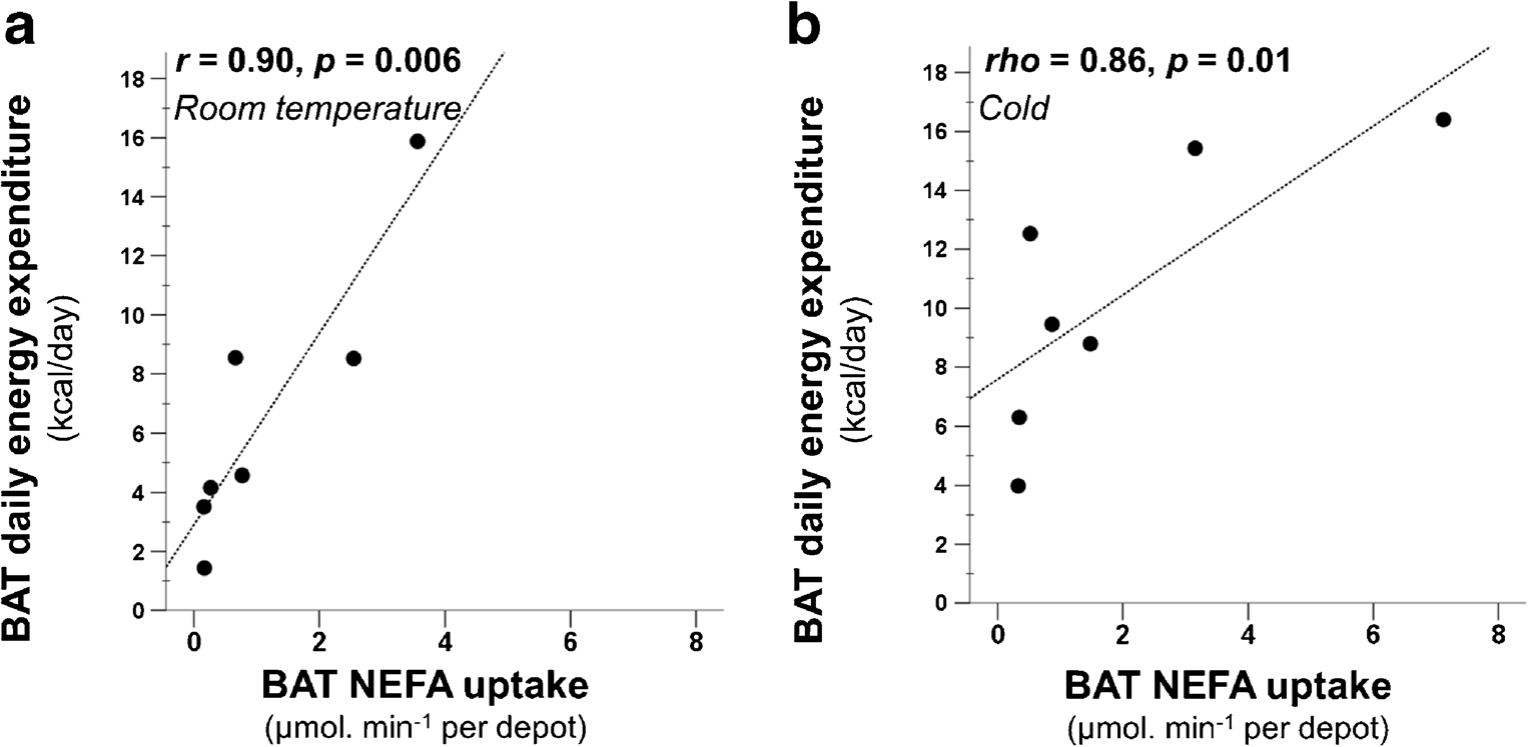
BAT-associated DEE correlated with BAT NEFA uptake both at room temperature (**a**) and also after cold stimulus (**b**). Abbreviation: NEFA, non-esterified fatty acid

Signifying tissue metabolism, BAT-associated DEE was in direct relationship with BAT blood flow both during RT (*r* = 0.97, *p* < 0.001, Fig. 6a) and cold (*r* = 0.79, *p* = 0.04, Fig. 6b). Moreover, it also had a direct relationship with BAT NEFA per depot uptake both during RT and under cold stress (RT: *r* = 0.90, *p* = 0.006; cold: *rho* = 0.86, *p* = 0.01, Fig. 7).

## Discussion

The present study addresses BAT-specific contribution to cold-induced thermogenesis (CIT), previously speculated to be primarily a BAT response in a number of reports [3, 7, 9, 22]. In addition, BAT substrate oxidative metabolism with particular reference to circulatory NEFAs is also addressed. Our study is one of the few attempts to evaluate oxygen consumption of BAT in humans using [^15^O]O_2_ PET imaging. A previous study by Muzik et al. [11] has shown an association between BAT oxygen consumption and semi-quantitative glucose uptake measure, SUVs. However, it has been suggested that the main substrate of activated BAT is fatty acid [10], and glucose is used as a secondary substrate. Therefore, it was critical to establish how much of the fatty acid uptake is associated with oxidative metabolism of BAT both at RT and during cold stimulus. One of the advantages of measuring oxygen consumption in BAT using [^15^O]O_2_ PET imaging is that it is a direct and non-invasive technique, which is not influenced by substrate availability and utilisation rate as might be the case with [^18^F]FDG and [^18^F]FTHA radiotracers [23]. This technique shows the overall oxidative metabolic rate of BAT, which also includes oxidation of all known possible substrates for BAT consumption, e.g. glucose, fatty acid, ketone bodies, or amino acids.

Based on our data, we estimated that under mild cold stimulation the BAT-associated DEE of the cervico-upper thoracic depot is approximately 10 ± 5 kcal/day which is close to the values reported earlier by Muzik et al. [11]. Whole-body EE increased significantly during cold stress; however, we did not observe any significant linear relationship between BAT DEE and whole-body EE which is indicative that the cervico-upper thoracic BAT does not contribute as a single and linear factor to whole-body EE. Change in BAT-associated DEE (4 ± 3 kcal/day) accounted for only 1 % of the total whole-body CIT (351 ± 372 kcal/day), while once DEE of cervico-thoracic muscles (in our FOV) was also taken into account they contributed 86 ± 68 kcal/day. However, the rest of the CIT could not be accounted for due to limited FOV of our PET scanner. The study by Blondin et al. [24] supports these findings, where it has been shown that the cold exposed skeletal muscles manifest more than 50 % of the total systemic glucose uptake compared to 1 % in BAT.

Acute cold stress in humans results in autonomic responses of cutaneous vasoconstriction, in order to limit heat loss, and activation of SNS [25]. Cutaneous vasoconstriction decreases skin temperature (Table 2), and elevation of circulatory catecholamine, as a consequence of SNS activation, triggers lipolysis in adipocytes. Increased lipolysis raises the plasma triglyceride levels (Table 2) likely to fuel NST [26]. Heat loss exceeding NST leads to an increase in shivering thermogenesis [25]. Therefore, in order to keep the subjects in the NST-zone, in our cooling protocol we increased the temperature of the cooling blanket once there were visual signs of shivering or the subject verbally reported shivering; however, based on our data it appears that shivering got activated on a microscale level in certain muscles before being observable or perceived by the individual. MRO_2_ in the deltoid and trapezius muscle did not increase significantly in our study, which suggests that shivering was not taking place in the appendicular skeletal muscles, and therefore, we deduce that possibly shivering perception in humans is only associated with shivering in the muscles of the appendages. Another explanation, other than the shivering for the increase in MRO_2_ in deep muscles, could also be the presence of mitochondrial uncoupling proteins as thermogenic agents in response to cold. These agents have been previously identified in rodent and human skeletal muscles [27–29]. Irrespective of the underlying mechanism of thermogenesis in skeletal muscles, our data suggests that the deep, centrally located cervico-thoracic muscles contribute to cold-induced thermogenesis along with BAT, and that they are a rather major contributor to CIT. Although, interestingly, the BAT mass correlated with CIT (Fig. 5), consistent with earlier findings [22], it appears that BAT possibly has an endocrine role in CIT, while the muscles surrounding BAT are the major contributor. Further studies that unravel secretory factors of BAT during cold stimulus, in conjunction with BAT oxygen consumption, are needed to draw concrete conclusions.

The strong positive correlation between BAT DEE and blood flow signifies the interdependence of both processes (Fig. 6). This may indicate that either UCP-1 mediated heat produced in BAT is distributed via circulation, or that the perfusion increases to provide oxygen and substrates for active BAT (Fig. 6). We further found that BAT-associated DEE is in correlation with its NEFA uptake both during RT and cold stress (Fig. 7). This suggests that BAT is also oxidizing substrates at RT although in a modest quantity compared to cold conditions, which signifies that BAT could already have ongoing thermogenesis at RT; however, it is not established how much of the UCP-1 protein in BAT mitochondria are uncoupled at RT. We also estimated that if the calculated fatty acids taken up via the blood stream in BAT, during cold stimulus, undergo complete oxidation they will amount to an average of 4.4 kcal/100 g/day (range 1.3–13.2 kcal/100 g/day). Comparing the values of both energy consumption following fatty acids complete oxidation and BAT-associated DEE, we observed a highly variable proportional contribution of circulatory NEFA towards the total BAT-associated DEE (14 % to 146 %, Supplementary Table 1). This variation was marginally linked to the quantity of stored triglycerides within BAT depots (measured as CT radiodensity in Hounsfield units), where less radiodense BAT depots (possessing more intracellular lipids) were found to require less contribution of NEFA from blood circulation (*r* = 0.72, *p* = 0.068, Supplementary Fig. 1). Other factors that may play a role in determining the quality and quantity of oxidised substrates could not be established with the current methods. Although it has been indicated that human BAT relies on the endogenous fatty acids following intracellular triglyceride breakdown [30]; there is currently no available tool that has the ability to measure the quantity, as well as the proportional contribution of these consumed lipids in vivo as a result of cold exposure. In our study we also could not determine whether circulatory NEFA taken up by BAT are directly consumed as a substrate for thermogenic respiratory chain reactions, or whether the NEFA replenish the intracellular triglyceride stores subsequent to endogenous production of fatty acids. Nevertheless, it is worth mentioning that energy content of fully oxidised NEFA taken up by BAT, reported here and previously [30], and glucose uptake [4, 7, 31] is of the same order of magnitude, although their proportional contribution during cold stimulus is yet to be determined.

The other limitations of our study include small number of study subjects which is due to the complexity of the radioactive oxygen PET scans. The complexity includes the production of the [^15^O]O_2_, the inhalation protocol, the PET image acquisition, and the kinetic modelling of this very rapidly defusing and decaying radiotracer. Moreover, relatively shorter half-life of ^15^O and the limited FOV of our scanner restricted assessment of possible BAT depots in further locations [32]; therefore, the present results may underestimate BAT contribution to whole body EE. Additionally, due to our aforementioned limitations, the relationship of metabolism in other organs to BAT oxidative metabolism could not be established.

## Conclusion

We conclude that cold stimulation in humans increases BAT-associated EE and whole-body EE; however, BAT is minor contributor to this whole-body cold-induced thermogenesis, while deeper centrally located neck muscles, along with the pectoral muscles are among the major contributors to thermogenesis. Moreover, in BAT, both during RT and cold stress, oxygen consumption is interlinked with the circulatory uptake of NEFA.

## Electronic supplementary material

Below is the link to the electronic supplementary material.

ESM 1 (PDF 164 kb)
