# Supplementary material for: Human brown adipose tissue [15O]O2 PET imaging in the presence and absence of cold stimulus
Source: Eur J Nucl Med Mol Imaging. 2016 Mar 19;43:1878–86. doi: 10.1007/s00259-016-3364-y (PMC4969352; doi:10.1007/s00259-016-3364-y)
Supplement: Supplementary file 1 — (PDF 164 kb) [file 259_2016_3364_MOESM1_ESM.pdf]

European Journal of Nuclear Medicine and Molecular Imaging

Supplementary Data

Human brown adipose tissue [^{15}O]O_2 PET imaging in presence and absence of cold stimulus

Mueez u Din, Juho Raiko, Teemu Saari, Nobu Kudomi, Tuula Tolvanen, Vesa Oikonen, Jarmo Teuho, Hannu T Sipilä, Nina Savisto, Riitta Parkkola, Pirjo Nuutila, Kirsi A. Virtanen

Corresponding author: Kirsi A. Virtanen, Ph: +358407626564, Fax: +35822318191, email: kirsi.virtanen@utu.fi

Methods

Production of tracers

Low-energy deuteron accelerator Cyclone 3 (Ion Beam Application Inc., Louvain-la-Neuve, Belgium) was used for the production of [^{15}O]O_2. [^{15}O] isotope was produced by the [^{14}N] (d,n) [^{15}O] nuclear reaction on natural nitrogen gas *(1)*. Radiowater [^{15}O]H_2O was produced using Hidex Radiowater Generator (Hidex Oy, Turku, Finland). Production of [^{18}F]FTHA has been described by Saari et al., *(2)*.

PET image reconstruction

All quantitative corrections were applied to PET image data, including detector normalization, dead-time, radioactive decay, randoms, attenuation and scatter. Images were reconstructed using iterative 3D-OSEM (GE Vue Point HD-S) reconstruction using 24 subsets and 2 iterations. All images were filtered using 6.4 mm Gaussian post-filter. Due to the presence of large in-FOV and out-FOV activity in the first frames in the [^{15}O]O_2 inhalation study, a clinically implemented scatter limit was applied (GE Quantitative Scatter Limit). The procedure is necessary to prevent scatter overestimation due to large in-FOV and out-FOV activity *(3)*.

Supplementary Table 1.				
**Study Subject**	**NEFA uptake rate (palmitic acid)**	**Energy Equivalent following complete circulatory NEFA oxidation***	**DEE from MRO_2**	**% of Energy from circulatory NEFA****
	**μmol/100g/min**	**kcal/100g/day**	**kcal/100g/day**	**percentage**
1	0.43	1.5	7.9	18.5
2	1.54	5.2	9.1	57.0
3	0.59	2.0	7.2	27.8
4	0.39	1.3	9.4	14.5
5	3.91	13.2	9.0	146.5
6	1.95	6.6	9.5	68.9
7	0.39	1.3	4.3	31.0

* Equation used for calculating energy equivalent

$$Energy\ Equivalent: palmitic\ acid\ (kcal/100g/day) = NEFA\ uptake\ rate\ (\mu mol/100g/min) \times 1440\ (min/day) \times 9.4\ (kcal/g) \times 256.42 \times 10^{-6}\ (g/\mu mol)$$

** Assuming complete oxidation of palmitic acid ([^{18}F]FTHA is palmitic acid based radiotracer)

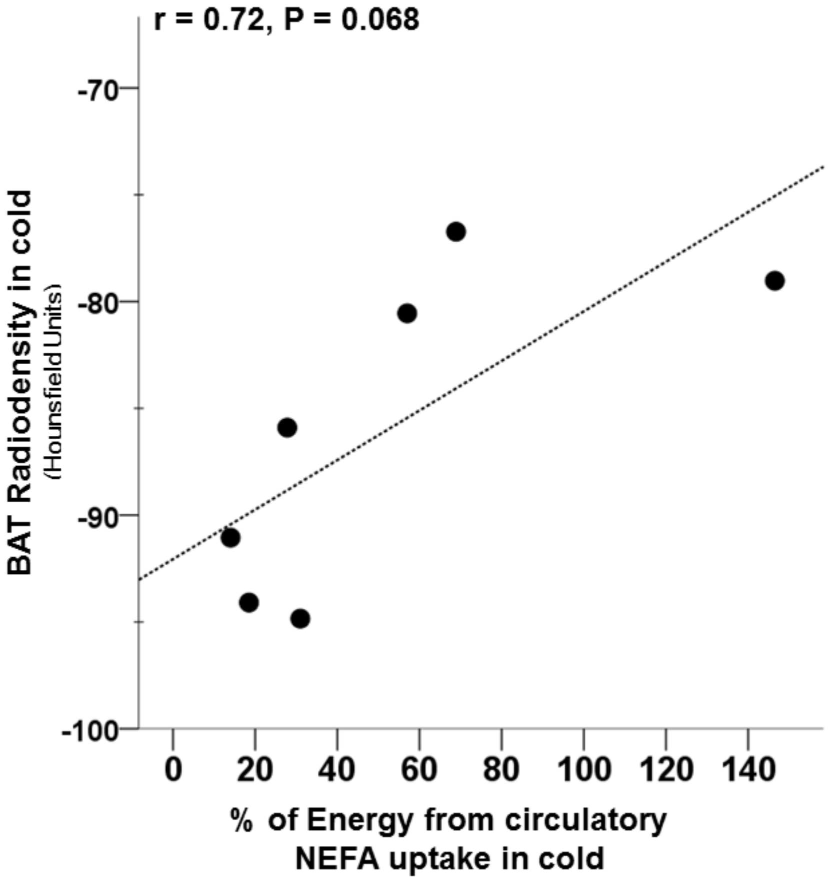

Supplementary Fig. 1 BAT with less radiodensity (possessing more stored lipids) uses less contribution of circulatory NEFA as energy substrate and vice versa (*r* = 0.72; *p* = 0.068).

Reference List

(1) Strijckmans K, Vandecasteele C, Sambre J. Production and quality control of 15O2 and C15O2 for medical use. *Int J Appl Radiat Isot*. 1985;36(4):279-283.

(2) Saari T. Impaired brown adipose tissue fatty acid metabolism in obese subjects [abstract]. *Diabetologia*. 2014;57(Suppl1).

(3) Hori Y, Hirano Y, Koshino K et al. Validity of using a 3-dimensional PET scanner during inhalation of 15O-labeled oxygen for quantitative assessment of regional metabolic rate of oxygen in man. *Phys Med Biol*. 2014;59(18):5593-5609.
